# The Academy for Multidisciplinary Neurotraumatology (AMN) Congress 2023: 20 Years of Excellence in Neurotraumatology

**DOI:** 10.25122/jml-2023-1021

**Published:** 2023-06

**Authors:** Stefana-Andrada Dobran, Alexandra Gherman, Dafin Muresanu

**Affiliations:** 1 RoNeuro Institute for Neurological Research and Diagnostic, Cluj-Napoca, Romania; 2 Sociology Department, Babes-Bolyai University, Cluj-Napoca, Romania; 3 Department of Neuroscience, Iuliu Hatieganu University of Medicine and Pharmacy, Cluj-Napoca, Romania

**Figure 1:**
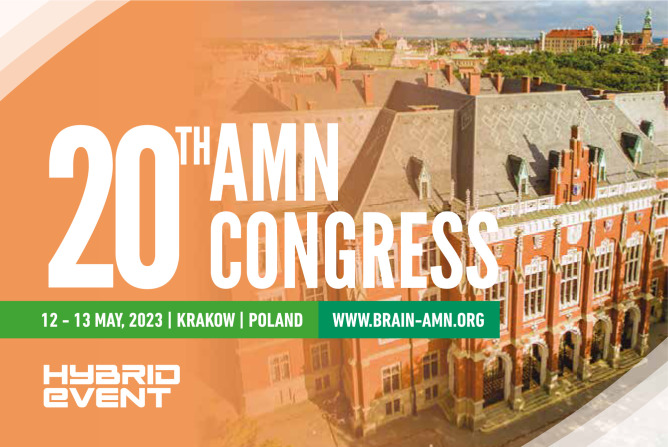
The 20^th^ Congress from the Academy for Multidisciplinary Neurotraumatology (AMN)

The 20^th^ edition of the Congress organized by the Academy for Multidisciplinary Neurotraumatology (AMN) took place in the spring of 2023. This significant milestone provided an opportunity to reflect on two decades of scientific advancements in the field of neurotrauma. Renowned speakers and participants from around the world gathered in the captivating city of Krakow, Poland, from May 12^th^ to May 13^th^, 2023 (Figure 1) and brought forward a dynamic focused on addressing the current and future challenges in neurotraumatology. Simultaneously, the Congress highlighted the advantages of engaging multidisciplinary approaches in overcoming the limitations encountered in the field. To foster scientific progress, the AMN Scientific Committee placed particular emphasis on exploring the latest developments in assisted rehabilitation and enhancing the quality of life for individuals with traumatic brain injuries (TBIs). The Congress employed a hybrid model to increase accessibility to information, extending its reach beyond geographical borders. This model enabled a large audience to explore the complexities of neurotraumatology with greater depth. The event was attended by over 250 participants from diverse nations, including Romania, Poland, Egypt, Azerbaijan, Uzbekistan, and Mexico.

The AMN actively strives for the advancement of neurotraumatology through various engagements, bringing upfront international scientific congresses to facilitate, encourage, and enhance knowledge and expertise exchange through comprehensive collaboration activities among experts. The Academy for Multidisciplinary Neurotraumatology makes the best use of its communication pillar that, over the decades, has developed into an acknowledged network of national and international associations, societies, and organizations involved in neurotraumatology. Furthermore, it is worth mentioning that the AMN is committed to delivering excellence in education by fostering cooperation with scientific academies, associations, societies, research institutions, and companies dedicated to neurotraumatology. The Academy boasts a global membership of over 600 individuals and maintains partnerships with prominent organizations such as the European Federation of Neurorehabilitation Societies (EFNR), World Federation for Neurorehabilitation (WFNR), the Foundation of the Society for the Study of Neuroprotection and Neuroplasticity (SSNN), European Academy of Neurology (EAN), European Society of Clinical Neuropharmacology (ESCNP), and the Foundation for the Study of Nanoneurosciences and Neuroregeneration. These collaborations enable the AMN to stay at the forefront of neurotraumatology research, teaching, and practical applications.

A teaching course on cognitive assessment after neurotrauma marked the beginning of the congress. The course was conducted by Prof. Nicole von Steinbüchel (Germany), the past President of the Academy for Multidisciplinary Neurotraumatology (AMN), who discussed the most relevant scientific scales in the domain of post-neurotrauma cognitive assessment.

The welcome address of the 20th AMN Congress was expressed by Prof. Dr. Dafin Muresanu (AMN Secretary General & EFNR President), Prof. Volker Hömberg (AMN Chairman of the Scientific Committee & WFNR President) and the president of the AMN, Prof. Johannes Vester.

The first day of Congress was built upon a four-session structure, each session focusing on different aspects of neurotrauma. The Presidential Session covered topics related to neurological rehabilitation, recent advancements in clinical research on traumatic brain injury (TBI), non-interventional comparative effectiveness in TBI, and the impact of sociodemographic, premorbid, and injury-related factors on post-traumatic stress, anxiety, and depression following TBI. Complementary to the first session, the AMN Presidential Panel entitled “The pitfalls of neurotrauma basic and clinical research", chaired by Michael Chopp (USA) and Dafin Muresanu (Romania), explored both the challenges and advancements of neurotrauma dynamics, with an emphasis on the value of multidisciplinary perspectives as a tool for broadening the field of neurotraumatology. The second session was focused on blood-brain barrier monitoring during acute ischemic stroke, the role of neurotrophic factors, the influence of endothelial cell-derived exosomes, and the effect of concussive head injury on Alzheimer's disease pathology. The third session marked a shift from the effects of neurotrauma to prehospital care for severe TBI patients and the use of multimodal approaches in ICU-based TBI treatment.

The closing fourth session of the day centered around diffuse axonal injury (DAI), endoscopic surgery for acute subdural hematoma, and surgical interventions for TBI. As such, expertise was illustrated on the duration of coma after DAI, the occurrence of persisting motor weakness, and time of recovery for patients with DAI as opposed to focal injuries; regarding research on acute subdural hematoma (ASDH), radiologic and clinical data were presented, converging to the conclusion that endoscopic subdural hematoma removal is, under certain conditions, an effective and safe surgical technique. Further on, going beyond the need for guidelines in neurotrauma, the time factor was highlighted as a central focus in neurosurgery, with “Time is Brain” surpassing all technological developments. Esteemed speakers present at the event included Johannes Vester (Germany), Dafin Muresanu (Romania), Volker Hömberg (Germany), Nicole von Steinbüchel (Germany), Sławomir Michalak (Poland), Michael Chopp (USA), Hari Shanker Sharma (Sweden), Helmut Trimmel (Austria), Klaudyna Kojder (Poland), Wojciech Dabrowski (Poland), Pieter Vos (The Netherlands), Se-Hyuk Kim (Korea), and Christian Matula (Austria), Razvan Chereches (Romania), Karin Diserens (Switzerland), Peter Lackner (Austria), Jongmin Lee (South Korea), Johannes Leitgeb (Austria), Marcin Michalak (Poland), Polona Pozeg (Switzerland), Katrin Rauen (Switzerland), and Andreas Winkler (Austria).

The second day of the congress commenced with a round table entitled “Real-World Evidence in TBI – Why Registries Matter”, a dynamic session that highlighted the significance of real-world data in TBI and the vital role of registries in neurotrauma management, pinpointing the latest developments and implementation stages at various country levels. The Patient Registry - Short Essential Neurotrauma (PRESENT) is an innovative initiative designed to provide comprehensive and multidisciplinary data regarding traumatic brain injury management and facilitate healthcare delivery across different countries. The registry also encourages long-term follow-up of patients. Marcin Michalak (Poland) presented the experience with the first 50 Polish patients included in the PRESENT registry, emphasizing its practical application. Furthermore, Peter Lackner (Austria) underlined the importance of neurotrauma registries in obtaining accurate assessments to further enhance patient care, and Razvan Chereches (Romania) discussed the piloting of a TBI registry in Moldova, Armenia, and Georgia. Following the presentations, the speakers shared interactive discussions with the audience. Session 5 of the event delved into post-TBI neurological assessments, highlighted the global burden of post-TBI depression, and emphasized the role of interdisciplinary neurotrauma management.

A stimulating discussion was brought forward by "The AMN Vision - Panel on How to Develop Multidisciplinary Teams in Clinical Routine. The NTSC Vienna concept" that pinpointed to the innovative Neurotrauma Treatment Simulation Center (NTSC) Program. The NTSC Program had its second edition on April 16-21, 2023 in Vienna, Austria, showcasing a novel approach to neurotrauma education and training. An innovative method in the field of neurotrauma, the program brings together specialists from various backgrounds, such as neurology, neurosurgery, trauma care, and anesthesiology, for a five-day training program in different medical locations. The panel, chaired by participants from the first NTSC edition, respectively Agata Andrzejewska (Poland) and Bassem Boulos (Egypt), featured the Austrian faculty members of the NTSC program, namely Christian Matula, Peter Lackner, Andreas Winkler, Johannes Leitgeb, and Helmut Trimmel. The special panel explored the concept of experiential learning and multidisciplinary teamwork promoted by the NTSC while placing under the loop the objectives of the program and the real-world impact it has on diagnosis, treatment, patient care, and follow-up.

The last session of the day focused on motor recovery after neurotrauma, psychiatric neurorehabilitation, and multimodal neuroimaging of covert consciousness. Once again, experience and knowledge were shared regarding specific challenges in neurorehabilitation, i.e., high incidence and prevalence of TBI, notable and permanent degree of disability, and multimorbidity in the elderly. With the concept of neuronal plasticity as the core scientific basis for neurorehabilitation, therapeutic interventions, such as Transcranial Magnetic Stimulation (TMS) and Transcranial Direct Current Stimulation (tDCS) are of top importance for restructuring neural networks.

**Figure 2:**
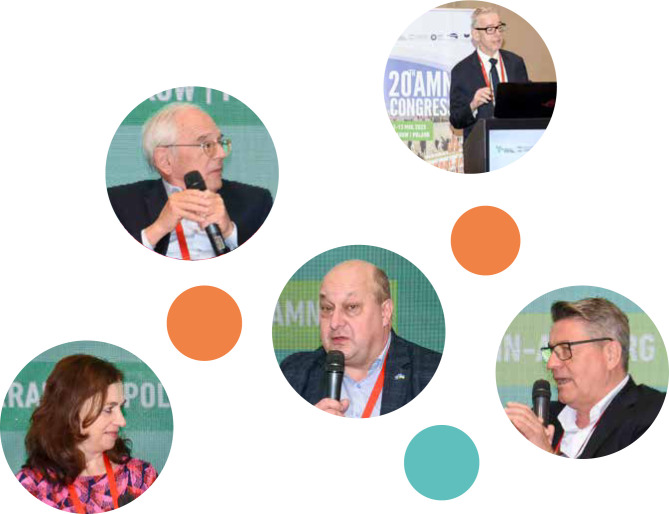
AMN Interview Series featuring Johannes Vester (upper right), Katrin Rauen (lower left), Andriy Huk (center), Michael Chopp (upper left), and Andreas Winkler (lower right).

The AMN conducted an insightful interview with the AMN President Johannes Vester, addressing key messages for the Congress participants and AMN members and future developments and projects of the Academy for Multidisciplinary Neurotraumatology. Further on, the AMN Interview Series (Figure 2) that concluded the congress represented a premiere and featured Michael Chopp (USA) being interviewed by Andreas Winkler (Austria) on advances in TBI neurosciences and Katrin Rauen (Switzerland) interviewing Andriy Huk (Ukraine) on the challenges of trauma care in Ukraine. All AMN interviews will be available in the very near future on the official AMN website at www.brain-amn.org and on YouTube.

**Figure 3:**
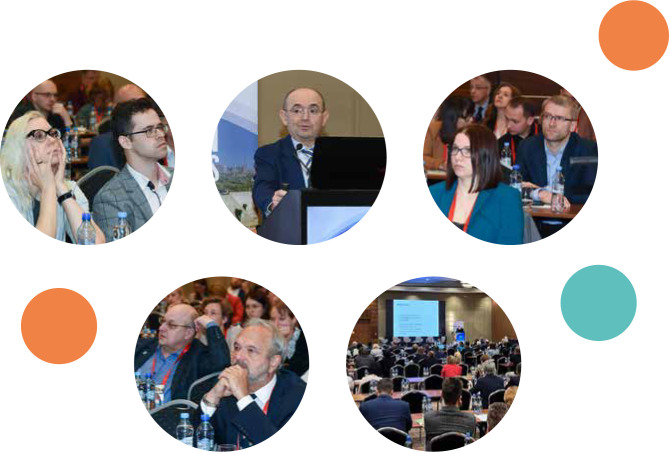
Participants and speakers from the 20^th^ AMN Congress

The 20^th^ edition of the AMN Congress (Figure 3) stands as proof of the unwavering commitment of the Academy to advancing neurotrauma research and improving patient care. Two decades of scientific pursuit showcase the progress in understanding and addressing the challenges in neurotrauma research and care. Focusing on a multidisciplinary approach, the congress explored projects such as the PRESENT registry and the NTSC program, that aim to transform neurotrauma education, research, and treatment. The Academy for Multidisciplinary Neurotraumatology plays a crucial role in enhancing the lives of individuals affected by traumatic brain injuries worldwide and sets the path for a future in which comprehensive and successful interventions result in better outcomes and enhanced quality of life for patients by pushing the boundaries of neurotrauma research and treatment.

**Figure 4:**
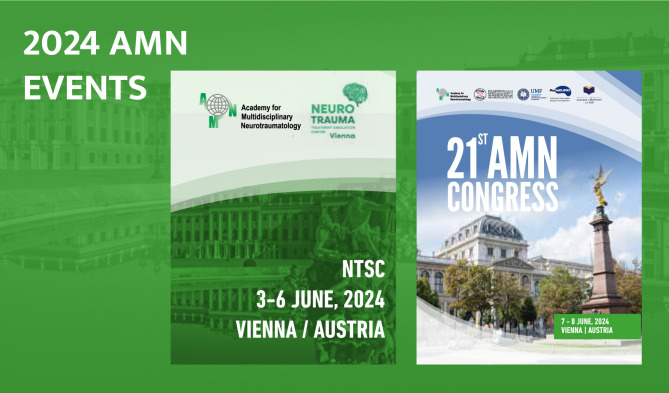
Announcement of the AMN 2024 Events

In 2024, the AMN will host two landmark events, the AMN Congress, between 3-6 June, and the 3rd edition of the Neurotrauma Treatment Simulation Center (NTSC), between 7-8 June (Figure 4). The events are set to encourage new perspectives in promoting innovation and multidisciplinary approaches, as well as addressing the gaps and limitations in the field of neurotraumatology. Join the AMN in the pursuit of improving research and education in the field of neurotrauma!

